# Brain-first versus body-first Parkinson’s disease: Differential findings on pupillary, brainstem and vagus sonography

**DOI:** 10.1007/s00415-026-13973-0

**Published:** 2026-07-02

**Authors:** Uwe Walter, Michael Batchakaschvili, Hanna Rebekka Kleinlein, Jakub Radziwon, Wiebke Hermann, Hartmut Walter, Alexander Storch, Matthias Löhle

**Affiliations:** 1 Department of Neurology, Rostock University Medical Center, Gehlsheimer Str. 20, 18147 Rostock, Germany; 2 Deutsches Zentrum für Neurodegenerative Erkrankungen (DZNE) Rostock/Greifswald, Rostock, Germany; 3 Department of Neurology, Neurodegenerative Diseases and Neuroimmunology, Faculty of Health Sciences with the Institute of Maritime and Tropical Medicine, Medical University of Gdańsk, Gdańsk, Poland

**Keywords:** Parkinson’s disease, Pupillary reflex, Substantia nigra, Transcranial ultrasound, Ultrasonography, Vagus nerve

## Abstract

**Background:**

In Parkinson’s disease (PD), two pathogenetic subtypes have been proposed: a ‘brain-first’, with α-synuclein pathology arising in one hemisphere and spreading secondarily to the peripheral autonomic nervous system, and a ‘body-first’ subtype, with the pathology originating in the enteric or peripheral autonomic nervous system and subsequently spreading symmetrically to the brain. To dissect these subtypes, we assessed the association between pupillary dysfunction, mesencephalic raphe and substantia nigra changes, vagus nerve atrophy and vagal electrocardiographic parameters in PD patients and controls.

**Methods:**

In this single-center cross-sectional study, we included 54 people with PD and 60 matched healthy controls. Participants underwent clinical assessments, electrocardiography, and sonographic measurements of substantia nigra echoic area, midbrain raphe echo-score and vagus nerve caliber. Dynamic ultrasound pupillometry was performed in drug-naïve de novo PD patients and matched controls. The brain-first and body-first subtypes were classified based on the REM-sleep Behavior Disorder Screening Questionnaire and gastrointestinal symptoms.

**Results:**

Vagal atrophy increased with disease duration and severity. During the first decade of motor disease, vagal atrophy and dysfunction occurred in body-first but not brain-first PD. Sympathetic pupillary innervation was reduced in de novo body-first but not brain-first PD patients. However, parasympathetic pupillary innervation was reduced in both subtypes at the de novo stage. Substantia nigra hyperechoic area was asymmetrical in brain-first but more symmetrical in body-first PD.

**Conclusions:**

Our findings support the concept of two subtypes of PD in which the mesencephalic and vagal parasympathetic systems are affected in opposite sequences. Ultrasonic and electrocardiographic examination could facilitate early subtyping.

**Supplementary Information:**

The online version contains supplementary material available at 10.1007/s00415-026-13973-0.

## Introduction

Parkinson’s disease (PD) has been proposed previously to comprise two pathogenetic subtypes: a ‘brain-first’ (top-down) type with α-synuclein pathology initially arising in the brain and secondarily spreading to the peripheral autonomic nervous system, and a ‘body-first’ (bottom-up) type with the pathology originating in the enteric or peripheral autonomic nervous system and then spreading to the brain [1, 2]. It has been suggested that brain pathology in the de novo stage is more symmetrical in the body-first PD subtype, but asymmetrical in the brain-first PD subtype [3]. There is now increasing evidence that pathological α-synuclein may originate in the gastrointestinal tract and ascend to the brain via the vagus nerve (VN), contributing to neurodegeneration [4–7]. Studies have demonstrated that patients who underwent complete vagotomy exhibit a reduced risk of developing PD, supporting the hypothesis of a gut-brain axis in disease onset [8, 9]. Furthermore, high-resolution ultrasound studies highlighted VN atrophy in PD and its relation to autonomic dysfunction, although the relationship with disease stage and subtype is yet unclear [10–14]. In addition to measuring the caliber of the VN, ultrasound can also be used to assess pupillary function in PD, as well as to detect changes in echogenicity in the midbrain raphe—which are related to serotonergic function—and in the substantia nigra (SN)—which are related to dopaminergic function [15–18]. Here we wanted to investigate whether rostral (mesencephalic non-vagal) and caudal (medullary vagal) brainstem structures are affected simultaneously or sequentially in PD. To this end, we investigated the relationship between pupillary dysfunction, changes in the mesencephalic raphe and SN and atrophy of the VN in PD patients versus controls and in ‘brain-first’ versus ‘body-first’ PD.

## Methods

### Study sample and design

**Fig. 1:**
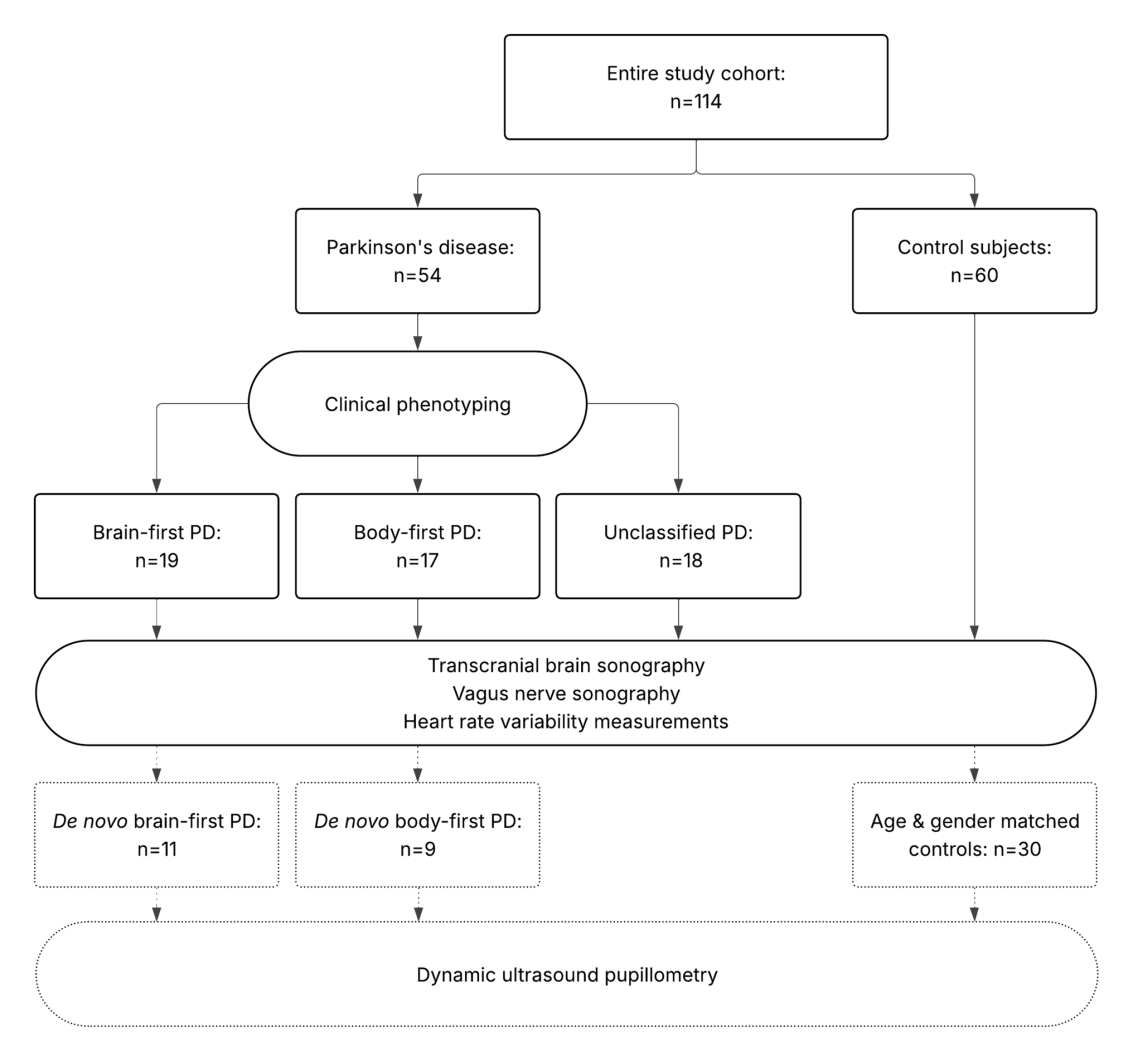
Study flowchart

We recruited 114 adult participants between September 2018 and February 2023 at the Department of Neurology of the University of Rostock, Germany. Fifty-four participants had PD (motor symptom duration: 7.2 ± 6.8 years, range 0.8–28 years; motor subtypes: 18 tremor dominant, 17 appendicular dominant, 1 rigidity dominant, 18 postural instability/gait difficulty) [19], and 60 were control subjects (Table 1**, **Fig. 1). The control group comprised healthy volunteers from our hospital staff or their relatives (n = 31) and patients with transient ischemic attack or minor stroke (n = 20), epilepsy (n = 5), or Bell’s paresis (n = 4). None of the controls and de novo PD patients had diabetes, impaired glucose tolerance, severe hypertension, coronary heart disease, neurodegenerative disease (except from PD in de novo patients), addiction diseases, regular intake of stimulants or sedatives, acute jetlag or shiftwork (during the past three months). The study participants underwent clinical, sonographic and neurophysiologic investigations usually on the same day or within ≤ 3 subsequent days. Participants were instructed to refrain from caffeine intake 12 h prior to pupillary sonography. The examiners performing the sonographic and neurophysiologic investigations were blinded to the clinical findings and to the ‘brain-first’ versus ‘body-first’ classification of PD patients. However, there was no complete blinding regarding group assignment (patients versus control group) for staff members and family members who were part of the control group.

**Table 1:** Demographic, clinical, and ultrasound findings in 54 PD patients and 60 controls

Feature	PD patients	Controls	P
Demographics			
Age (years), mean ± SD	68.7 ± 9.4	66.0 ± 8.7	0.11 ^a^
Gender, female/male (N)	21/33	25/35	0.90 ^b^
PD duration (motor onset, years)	7.2 ± 6.8		
Clinical scores			
MDS-UPDRS part III, median [IQR]	34 [24, 46]	0 [0, 4]	< 0.001 ^c^
NMSQ sum score, median [IQR]	9 [5, 14]	3 [2, 5]	< 0.001 ^c^
NMSS sum score, median [IQR]	42 [22, 83]	12 [3, 20]	< 0.001 ^c^
NMSS domain 1 (orthostatic hypotension)	0 [0, 3]	0 [0, 1]	0.041 ^c^
NMSS domain 6 (gastrointestinal tract)	2 [0, 12]	0 [0, 9]	< 0.001 ^c^
NMSS domain 7 (urinary function)	4 [0, 20]	1 [0, 6]	0.009 ^c^
NMSS domain 8 (sexual function)	0 [0, 0]	0 [0, 0]	0.46 ^c^
Olfactory testing			
SS-16 score, median [IQR] ^d^	7 [4, 9]	11 [10, 13]	< 0.001 ^c^
Heart rate variability (vagal component)			
RMSSD (ms), median [IQR] ^e^	18 [0, 28]	33 [19, 51]	< 0.001 ^c^
Sonography findings			
SN echoic area (mm^2^) ^f^	0.31 ± 0.09	0.18 ± 0.07	< 0.001 ^a^
SN, normal/hyperechoic (N) ^f^	9/44	49/10	< 0.001 ^g^
SN echoic area, asymmetry index ^h^	0.40 ± 0.26	0.36 ± 0.27	0.42 ^a^
SN echoic area, symmetric/asymmetric (N) ^h^	31/22	36/23	0.73 ^b^
Midbrain raphe echo score, median [IQR] ^h^	3 [3]	3 [3]	0.68 ^c^
Pupil diameter at baseline (mm) ^i^	3.50 ± 0.85	3.81 ± 0.92	0.10 ^a^
Pupil constriction velocity (mm/s) ^i^	2.23 ± 0.70	2.62 ± 0.91	0.020 ^a^
Pupil re-dilation velocity (mm/s) ^i^	0.97 ± 0.40	0.98 ± 0.36	0.94 ^a^
Vagus nerve CSA (mm^2^) ^j^	1.54 ± 0.55	1.79 ± 0.49	0.013 ^a^

CSA: cross-sectional area; MDS-UPDRS III: motor part of the MDS-sponsored Unified PD Rating Scale; NMSQ: Non-Motor Symptoms Questionnaire for PD; NMSS: Non-Motor Symptoms Score for PD; RMSSD: root mean square of successive differences of R-R intervals on electrocardiogram with 0.2-Hz metronom-guided breathing; SN: substantia nigra; SS-16: 16-item Sniffin’ Sticks test

^a^ *t*-test, two-sided ^b^ *χ*^2^ test ^c^ Mann–Whitney *U* test

^d^ assessed in 51 PD patients and 30 controls ^e^ assessed in 41 PD patients and 40 controls

^f^ larger of bilateral measures, SN bilaterally assessable in 53 patients and 59 controls

^g^ Fisher’s exact test ^h^ for details, see text ^i^ assessed in 20 de novo PD patients and 30 controls

^j^ individual mean of bilateral measures, VN CSA bilaterally assessable in 52 patients and 60 controls

The study was approved by the ethics committee of the Medical Faculty at Rostock University (identifier: A2018-0085) in accordance with the policies and ethical principles of the Declaration of Helsinki. Written informed consent was obtained from each participant.

### Clinical assessments

The severity of PD motor symptoms was evaluated with the Movement Disorder Society revised version of the Unified PD Rating Scale (MDS-UPDRS) part III [20]. Non-motor symptoms were assessed in all participants using the validated German versions of the PD Non-Motor Symptoms Questionnaire (NMSQ) and Non-Motor Symptoms Scale (NMSS) [21]. In the 34 PD patients and 30 controls who did not undergo pupillary sonography the self-perceived degree of over-sensitivity to bright light was assessed on item 19 of the Scales for Outcomes in PD—Autonomic Dysfunction (SCOPA-OUT) [22], representing an indirect measure of pupillomotor (pupil constriction) function [23]. Participants underwent a standardized psychophysical olfactory test, the 16-item smell identification test from Sniffin’ Sticks (SS-16; Burghart Messtechnik, Wedel, Germany) [24]. An SS-16 score < 10 was regarded to indicate hyposmia since this cut-off value has been reported to discriminate best between PD and non-PD subjects [25]. Though the diagnostic gold standard of REM-sleep behavior disorder (RBD) remains polysomnography [26], the RBD Screening Questionnaire (RBDSQ) has been demonstrated to provide high positive predictive value for RBD [27, 28]. We here applied a classification of probable RBD (pRBD) by the criterion RBDSQ score ≥ 6 as used in previous studies [29–31]. Since a lower sensitivity of the RBDSQ ≥ 6 criterion for the detection of polysomnography-proven RBD has been shown in de-novo PD patients, we applied in de-novo PD patients the criterion of RBDSQ score ≥ 6 and/or RBDSQ item 6 score ≥ 1 [32].

### Classification of brain-first vs body-first PD

The discrimination of brain-first versus body-first PD is so far based on the hypothesis of different α-synuclein pathology propagation based on clinical, multimodal functional imaging and postmortem histological findings [2, 3, 33]. A core clinical feature of body-first PD is a reliable history of RBD symptoms clearly antedating the onset of motor symptoms [33]; therefore video-polysomnography proven RBD, or questionnaire proven pRBD has been used as the classifier between body-first and brain-first PD in earlier studies [2, 29, 31]. On the other hand, in the body-first subtype α-synuclein pathology is present early in the enteric nervous system, and gastro-intestinal symptoms are frequent [29, 33]. In order to avoid misclassification we here included patients only with motor symptom duration < 10 years, and applied the following combined criterion for body-first PD: (i) presence of pRBD (see above), with onset of RBD symptoms prior to onset of motor symptoms, in combination with NMSS gastrointestinal subscore ≥ 2 and/or NMSS sum score ≥ 25, or (ii) if no RBD history was evident, NMSS gastrointestinal subscore ≥ 8, with onset of gastro-intestinal symptoms prior to onset of motor symptoms. Patients with motor symptom duration < 10 years who did not meet the combined criterion for body-first PD were classified as having brain-first PD, provided they had an RBDSQ score of ≤ 3 [31]. The remaining patients were categorized as “unclassified,” regardless of the duration of their motor symptoms.

### Pupillary sonography

**Fig. 2:**
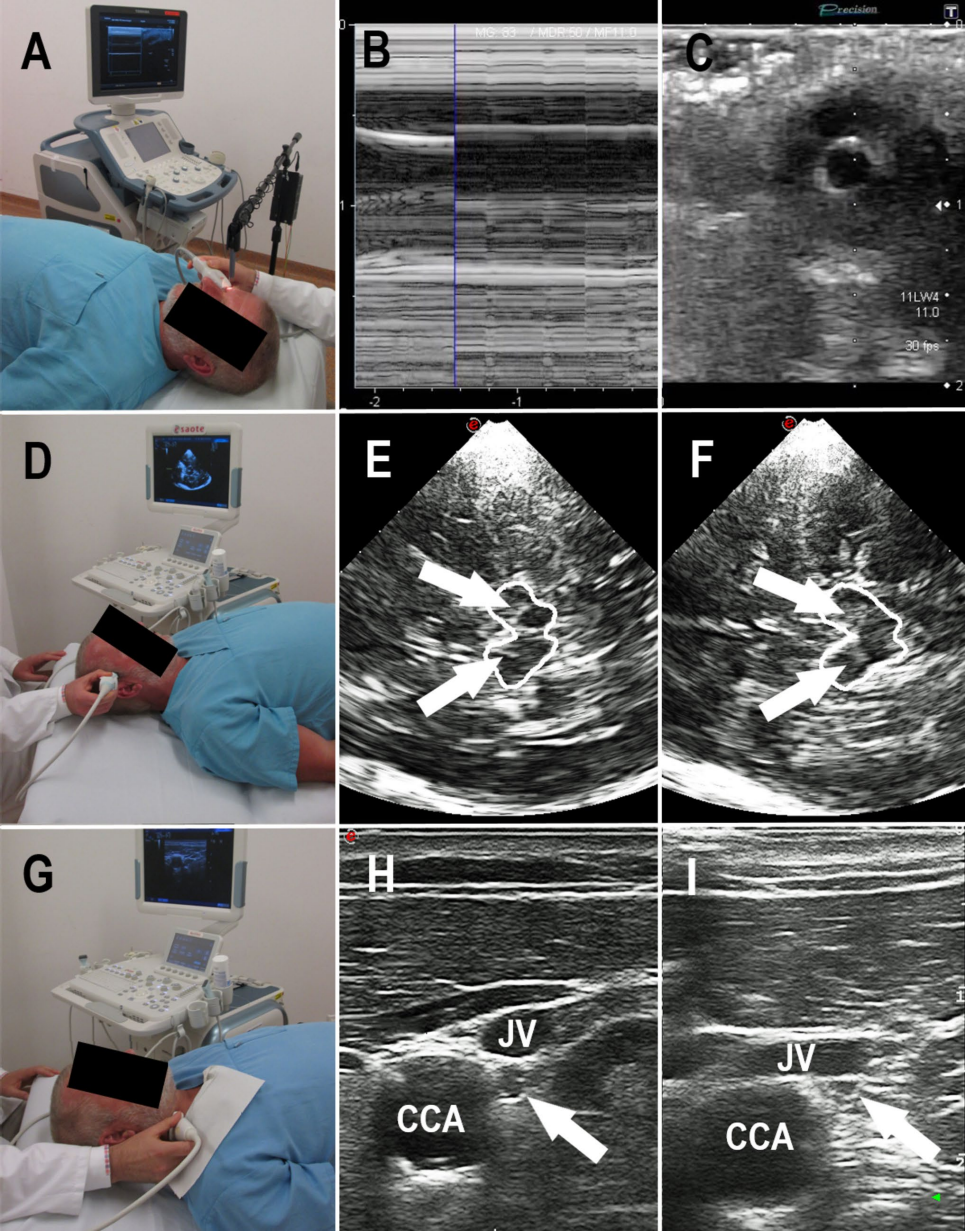
Illustration of ultrasonography of pupillary reflex, brainstem structures and vagus nerve. A) Positioning of the subject, the ultrasound probe, and the eye illumination device for dynamic ultrasound pupillometry (see text for details). B) Ultrasound image in motion mode (M-mode) showing the temporal progression of pupillary constriction with the penlight turned on. The blue vertical line marks the point of maximum pupillary constriction, which corresponds to the pupillogram shown in (C). C) Coronal sonogram of the pupil in brightness mode (B-mode) at the moment of its maximum constriction with the penlight turned on. The dotted vertical line marks the section used for the M-mode image shown in (B). D) Positioning of the subject and the ultrasound probe for transcranial sonography of the midbrain structures. E) Axial sonogram of the brain at the level of the midbrain. The butterfly-shaped midbrain is circled for better visualization. Note the asymmetric increase in echogenicity of the substantia nigra (arrows), which is typically observed in patients with the brain-first subtype of PD. F) Axial sonogram of the brain at the level of the midbrain. The midbrain is circled for better visualization. Note the symmetrical increase in echogenicity of the substantia nigra (arrows), which is typically observed in patients with the body-first subtype of PD. G) Positioning of the subject and the ultrasound probe for sonography of the mid-cervical vagus nerve. H) Axial neck image showing the common carotid artery (CCA), the internal jugular vein (JV), and the vagus nerve (arrow). Note the normal caliber of the vagus nerve, which is typically found in patients with the brain-first subtype of PD. I) Axial neck scan showing the common carotid artery (CCA), the internal jugular vein (JV), and the vagus nerve (arrow). Note the reduced caliber of the vagus nerve, which is typically found in patients with the body-first subtype of PD, particularly in advanced stages of the disease

Twenty de novo, dopaminergic drug-naïve PD patients (9 women; age 64.5 ± 9.2 years) und 30 age- and sex-matched controls (14 women; 64.8 ± 6.4 years) underwent ultrasonographic dynamic pupillometry. We excluded individuals suffering from pathological eye conditions, or diagnosed with conditions (diabetes mellitus), or treated with concomitant medication (anticholinergics, cholinesterase inhibitors, and glaucoma treatment), that could interfere with the pupil size evaluation. All assessments were performed by the same experienced investigator (UW) with a Toshiba Xario ultrasound system (Canon, Japan) equipped with a 7.5-MHz linear-array transducer (PLT-704SBT). The input of the ECG adapter cable was used to connect a custom-made electronic device to control the switching on and off of a commercially available medical penlight (for electronic circuit diagram, see Supplementary Fig. S2), which allowed for the indication of light switches on the ultrasound screen (B-mode, M-mode). A foot switch was used to start the light, and the time of light on was set at automatic stop (light off) after 1.7 s. The ultrasonic recordings were conducted sequentially on the right and the left eye of the lying subjects in a quiet and fully darkened room after at least 5 min of dark adaptation. According to current recommendations on orbital insonation, the mechanical index was adjusted below 0.26 and the total examination time was kept as short as possible. The probe was placed below the orbit on top of the zygomatic bone while applying gentle pressure and then tilted downwards to approximately 45° in order to insonate the iris plane [34]. At this level, the pupil presents as an anechoic round structure surrounded by an hyperechoic ring, the iris. In order to achieve a stable image of the pupil, participants were instructed to look upwards. After clear simultaneous B-mode and M-mode visualization, the ipsilateral pupillary light reflex (constriction after switching on the light, re-dilation after switching off the light) was recorded, with the penlight of fixed intensity (8 lumens) placed 2 cm in front of the subject’s closed eye (Fig. 2A). Custom software coded in Matlab (version 7.12.0, Mathworks, Massachusetts USA) controlled stimulus presentation, pupil recording and analysis. Baseline pupil width at rest (before light exposure), pupil constriction amplitude, maximum pupil constriction velocity, and maximum pupil re-dilation velocity were analysed by a rater (MB) who was blinded to the category (brain-first, body-first) of PD patients. We used the maximum velocities (mm/s), since among the pupillometric parameters, maximum constriction velocity is considered to be the most robust measure for detecting parasympathetic dysfunction [35, 36]. The asymmetry index (AI) of individual right and left measures (pupil width at rest; constriction velocity) was calculated using as: AI = *abs* (Mr – Ml)/((Mr + Ml)/2), where “M” denotes the respective measure for the right (Mr) or left (Ml) eye and “*abs*” denotes the absolute value, ensuring that the index value is always positive.

### Midbrain sonography

Transcranial sonography (TCS) of midbrain was done by an experienced sonographer (UW) in all participants through the preauricular acoustic bone windows using a MyLabTwice ultrasound system (Esaote S.p.A., Italy) equipped with a 2.5-MHz phased-array transducer (PA240). The ultrasound system settings were as follows: view 3, size of aperture 89°, dynamic range 6, dynamic compression 2, persist 7, enhance 3, density 2, focuses 1, gray map 5, mechanical index 1.0 [37]. SN ipsilateral to insonation was assessed from both sides. Planimetric measurements of substantia nigra echogenic size were performed in all study participants on axial scans automatically after manually circling the outer circumference of the echoic area in the anatomic region of substantia nigra (Fig. 2B). With the ultrasound system and settings applied, echoic areas of < 0.24 cm^2^ are classified as normal, areas of ≥ 0.24 cm^2^ representing upper 25% percentile in normal population as hyperechoic, and areas of ≥ 0.30 cm^2^ representing upper 10% percentile in normal population as markedly hyperechoic [37]. The asymmetry index (AI) of individual right and left echoic areas was calculated using as: AI = *abs* (SNr – SNl)/((SNr + SNl)/2), where “SN” denotes the echoic area of substantia nigra for the right (SNr) or left (SNl) hemisphere and “*abs*” denotes the absolute value, ensuring that the index value is always positive. Higher AI values indicate greater asymmetry between hemispheres. Echogenicity of the mesencephalic raphe was rated on a three point scale using the highly echoic red nucleus as a reference point: 1 = raphe not visible, 2 = slightly echoic or interrupted raphe, 3 = normal, highly echoic raphe [15]. The larger score of bilateral assessments was used for further analysis.

### Vagus nerve sonography

A MyLabTwice ultrasound system (Esaote S.p.A., Italy) equipped with a 15.0-MHz linear-array transducer (LA435) was applied. Bilateral VN’s were scanned in the axial view as reported earlier in detail [38]. All measurements were performed by one of two well-trained sonographers (RK, UW). To capture the VN, the probe was placed at the mid-cervical level (at the level of thyroid cartilage (Fig. 2C)). For measurements of cross-sectional area (CSA), the target nerve was positioned in the center of the image, at a position where it had a nearly elliptic cross-sectional shape. For the axial transection, the longest cross-sectional diameter *a* of the nerve and the diameter *b* perpendicular to *a* were measured separately (within the hyperechoic epineural rim of the nerve), and the elliptic CSA (in mm^2^) was calculated off-line according to the formula: CSA = *a*·*b*·π/4. A high interrater correlation between investigators independently performing the VN imaging and measurement has been shown earlier (Pearson correlation, r = 0.88) [39]. Here, we assessed the interrater validity of VN CSA measures for using the same unannotated axial VN sonograms; for this, anonymized VN sonograms were reviewed by a second reader (JR) blind to the clinical data, who independently identified the VN and executed the CSA measurements.

### Heart rate variability

To obtain standard time-domain heart rate variability parameters, we performed a short bedside analysis of electrocardiogram in the PD patients and age-matched control subjects. Following 5 min of inactivity in a supine position, 5-min resting 4-lead electrocardiogram (aV_R_, aV_L_, N, aV_F_) was recorded during daylight hours in a non-fasting state with 0.2-Hz metronom-guided breathing using a neurophysiologic workstation (Keypoint G4; Natus Europe GmbH, Planegg, Germany) [40]. R-R intervals were automatically detected and visually inspected; records with atrial fibrillation or ectopic beats were not used for the analysis. The root mean square of successive differences of R-R intervals (RMSSD) was calculated as a parameter reflecting parasympathetic (vagal) innervation [41].

### Statistical analyses

Normally distributed variables were compared with the two-sided *t*-test, and categorical variables with the *χ*^2^ test and Fisher’s exact test. Shapiro–Wilk and Levene’s tests were performed to evaluate normality and homogeneity of variance, respectively; non-normally distributed variables are reported as median [interquartile range] and compared using the Kruskal–Wallis and Mann–Whitney *U* tests. The Pearson correlation test was used to assess interrater reliability of sonographic measures. The Spearman correlation test was used to compare sonographic measures with demographic and clinical parameters; since ten different parameters (PD motor symptom duration, MDS-UPDRS-3 score, NMSQ sum score, NMSS sum score, NMSS gastric subscore, RMSSD, VN CSA, Baseline pupil width, pupil constriction velocity, pupil re-dilation velocity) were tested in the correlation analyses, a Bonferroni correction was applied, with p < 0.005 indicating significance. In group comparisons, a two-sided p value < 0.05 was regarded as statistically significant. Receiver operating characteristic (ROC) curves were plotted to assess the value of sonographic und electrophysiologic findings for the indication of body-fist subtype PD in de-novo patients. Optimum cut-off values were estimated by calculation of the Youden Index *J*. Statistical analyses were performed with IBM SPSS Statistics software (version 30.0).

## Results

### Study participants

Demographic and clinical findings are shown in Table 1 and Supplementary Table S1. Of the 54 PD patients, 19 fulfilled the pre-defined clinical criteria of brain-first PD, and 17 the criteria of body-first PD (Table 2). Of these classified PD patients, 20 were de novo, dopaminergic drug-naïve patients (motor symptom duration: 1.5 ± 0.6 years; Supplementary Table S2). Motor subtypes did not differ between de novo brain-first PD patients (4 tremor dominant, 7 appendicular dominant) and de novo body-first PD patients (5 tremor dominant, 3 appendicular dominant, 1 postural instability/gait difficulty; *χ*^2^ test, p = 0.28).

**Table 2:** Findings in 19 brain-first vs. 17 body-first PD patients

Demographics			
Feature	Brain-first PD	Body-first PD	P
Age (years), mean ± SD	65.9 ± 8.9	68.1 ± 9.1	0.48 ^a^
Gender, female/male (N)	7/12	6/11	1.0 ^b^
PD duration (motor onset, years)	3.0 ± 2.6	3.2 ± 2.9	0.78 ^a^
Clinical scores			
MDS-UPDRS part III, median [IQR]	30 [21, 38]	38 [26, 46]	0.079 ^c^
NMSQ sum score, median [IQR]	5 [4, 9]	9 [6, 15]	0.013 ^c^
NMSS sum score, median [IQR]	21 [12, 35]	52 [28, 101]	0.001 ^c^
NMSS domain 1 (orthostatic hypotension)	0 [0, 3]	0 [0, 2]	0.51 ^c^
NMSS domain 6 (gastrointestinal tract)	0 [0, 0]	8 [4, 16]	< 0.001 ^c^
NMSS domain 7 (urinary function)	2 [0, 9]	9 [4, 24]	0.036 ^c^
NMSS domain 8 (sexual function)	0 [0, 0]	0 [0, 4]	0.005 ^c^
Olfactory testing			
SS-16 score, median [IQR] ^d^	9 [4, 12]	7 [4, 7]	0.025 ^c^
Heart rate variability (vagal component)			
RMSSD (ms), median [IQR] ^e^	28 [22, 32]	13 [0, 22]	0.006 ^c^
Sonography findings			
SN echoic area (mm^2^) ^f^	0.30 ± 0.10	0.30 ± 0.08	0.81 ^a^
SN, normal/hyperechogenic (N) ^f^	5/14	2/14	0.41 ^b^
SN echoic area, asymmetry index ^g^	0.50 ± 0.28	0.32 ± 0.22	0.040 ^a^
SN echoic area, symmetric/asymmetric (N) ^g^	7/12	14/2	0.005 ^b^
Midbrain raphe echo score, median [IQR] ^g^	3 [3]	3 [3]	0.80 ^c^
Pupil diameter at baseline (mm) ^h^	3.60 ± 0.94	3.37 ± 0.74	0.43 ^a^
Pupil constriction velocity (mm/s) ^h^	2.25 ± 0.70	2.20 ± 0.72	0.85 ^a^
Pupil dilation velocity (mm/s) ^h^	1.02 ± 0.45	0.91 ± 0.32	0.38 ^a^
Vagus nerve CSA (mm^2^) ^i^	1.89 ± 0.59	1.43 ± 0.41	0.011 ^a^

CSA: cross-sectional area, MDS-UPDRS III: motor part of the MDS-sponsored Unified PD Rating Scale, NMSQ: Non-Motor Symptoms Questionnaire for PD, NMSS: Non-Motor Symptoms Score for PD, RMSSD: root mean square of successive differences of R-R intervals on electrocardiogram with 0.2-Hz metronom-guided breathing, SN: substantia nigra, SS-16: 16-item Sniffin’ Sticks test

^a^ *t*-test, two-sided

^b^ Fisher’s exact test

^c^ Mann–Whitney *U* test

^d^ assessed in 19 brain-first & 15 body-first PD patients. ^e^ assessed in 14 brain-first & 15 body-first PD patients

^f^ larger of bilateral measures, SN bilaterally assessable in 19 brain-first & 16 body-first PD patients

^g^ for details, see text

^h^ assessed in 11 brain-first & 9 body-first de novo PD patients

^i^ individual mean of bilateral measures in 19 brain-first & 16 body-first PD patients

### Pupillary sonography

**Fig. 3:**
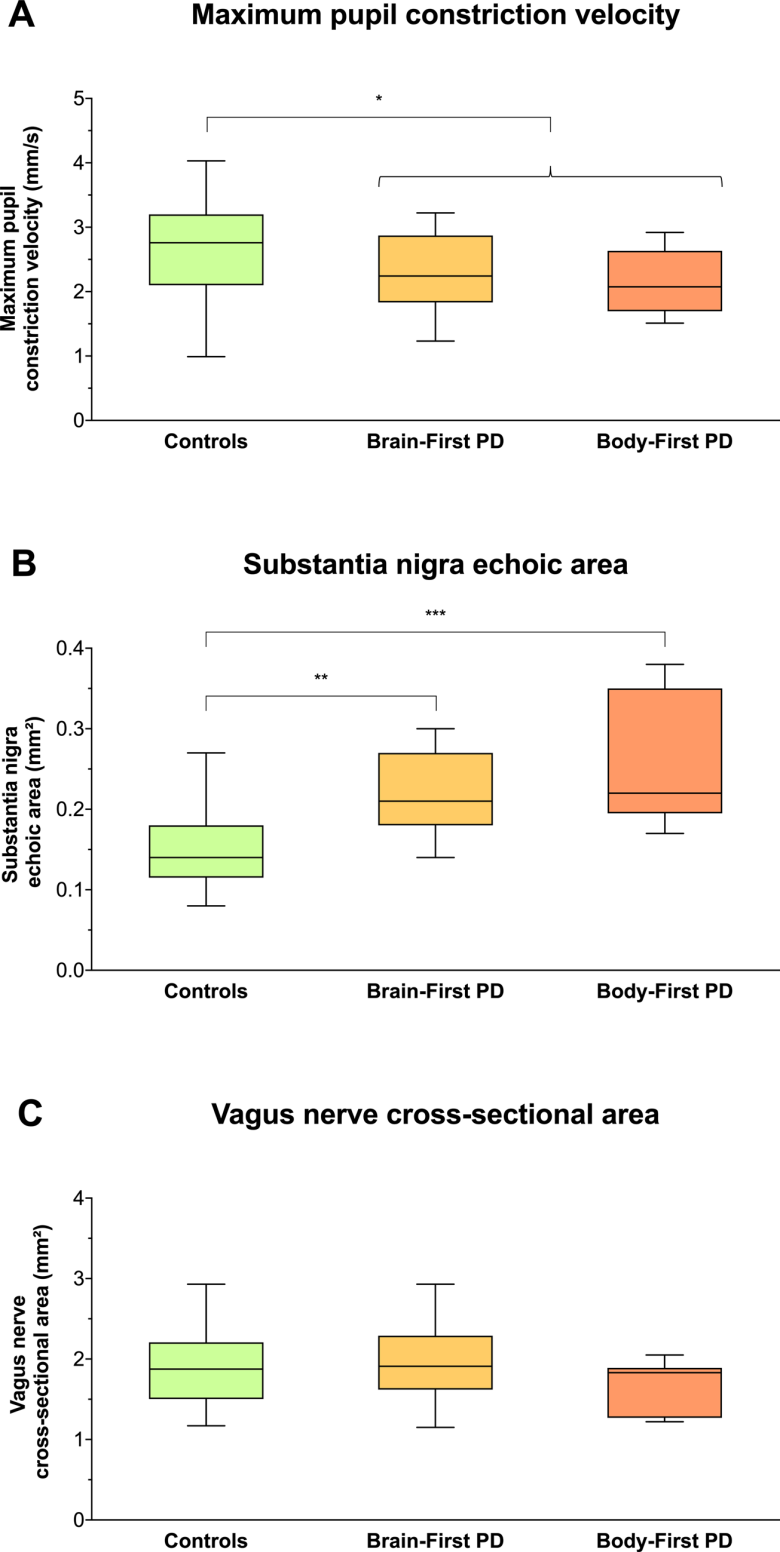
Box-and-whisker plots showing the ultrasonographic findings in the control group (n = 30), in de novo patients with brain-first PD (n = 11), and in de novo patients with body-first PD (n = 9). A) Maximum pupil constriction velocity did not differ between patients with brain-first PD and those with body-first PD; however, it was reduced in the combined group of patients with de novo PD compared with age-matched controls (* Mann–Whitney *U* test, p < 0.05). B) The echoic area of the substantia nigra did not differ between patients with brain-first PD and patients with body-first PD; however, it was enlarged in both patient groups compared to age-matched controls (** Kruskal–Wallis test, p < 0.005, *** p < 0.001). C) The cross-sectional area of the vagus nerve (individual mean calculated from measurements on both sides) did not differ either among the groups of patients with de novo PD or between these patient groups and age-matched control subjects

Pupillary sonography (Fig. 2A) was performed in 20 de novo (drug-naïve) PD patients and 30 age-matched controls. The baseline pupil diameter tended to be smaller in body-first PD patients vs. controls (3.37 ± 0.74 mm vs. 3.81 ± 0.92 mm, *t*-test, p = 0.055), but not in brain-first PD patients vs. controls (3.60 ± 0.94 mm vs. 3.81 ± 0.92 mm, p = 0.38). Individual asymmetry indices of pupil size did not differ between patients and controls (p = 0.17) nor between brain-first and body-first PD patients (p = 0.40). Baseline pupil diameter correlated with pupil re-dilation velocity (patients only [n = 38]: Spearman correlation, r = 0.64, p < 0.001; 95%CI: 0.39–0.80; all participants: r = 0.72, p < 0.001; 95%CI: 0.60–0.81). Pupil re-dilation velocity did not differ between patients and controls, nor between body-first and brain-first PD patients (Tables 1, 2). Pupil constriction velocity was reduced in PD patients compared to controls (2.23 ± 0.70 mm/s vs. 2.62 ± 0.91 mm/s, *t*-test, p = 0.020), but did not differ between body-first and brain-first PD patients (p = 0.85; Fig. 3A, Supplementary Fig. S1**)**. Individual asymmetry indices of pupil constriction velocity did not differ between patients and controls (p = 0.36) nor between brain-first and body-first PD patients (p = 0.29). Pupil width at rest, pupil dilation velocity, and pupil constriction velocity did not correlate with motor symptom duration or severity in the de novo PD group studied here with pupillary sonography.

### Midbrain sonography

Transcranial sonography of midbrain structures (Fig. 2B) was feasible bilaterally in 53 patients and 59 controls, only unilaterally successful (right side) in one PD patient, and unsuccessful in one control due to bilaterally insufficient bone windows. SN echogenic sizes were larger in patients than in controls (Table 1, Supplementary Table S2, Fig. 3B). SN echogenic sizes were unrelated to VN CSA’s and measures of pupillary function in controls and patients. SN asymmetry indices were smaller in body-first than in brain-first patients (Table 2). A pronounced asymmetric SN echogenicity (defined by asymmetry index > 0.4) was present in 12 (63%) of 19 brain-first but only in two (12%) of 16 body-first PD patients (Fisher’s exact test, p = 0.005). This finding did not change if analysis was restricted to de novo patients (64%, 11%, p = 0.028). Compared to controls, pronounced asymmetric SN echogenicity tended to be more frequent in brain-first PD patients (p = 0.069) but not in body-first PD patients. There was no side-dominance of pronounced asymmetric SN echogenicity in brain-first PD patients (right-sided dominance: n = 5, left-sided dominance: n = 7; Fisher’s exact test, p = 1.0), nor in body-first PD patients (n = 1, n = 1; p = 1.0). Midbrain raphe echogenicity did not differ between patients and controls, nor between brain-first and body-first PD patients (Tables 1, 2).

### Vagus nerve sonography

**Fig. 4:**
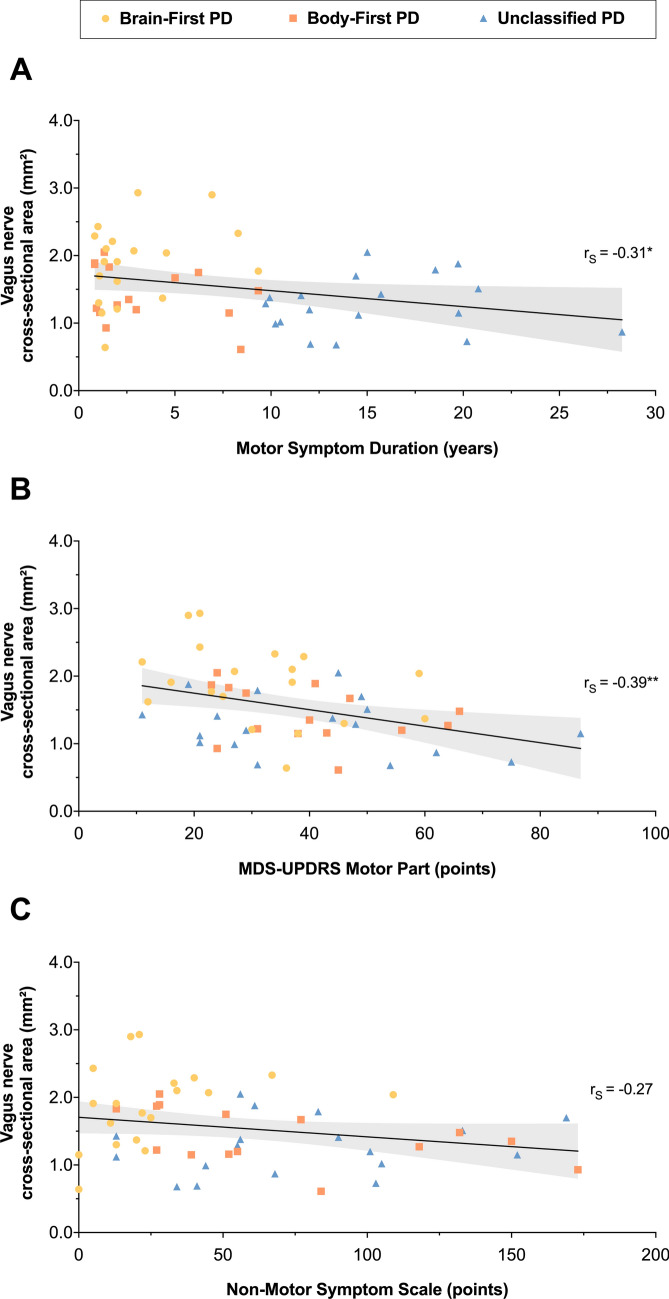
Graphs illustrating the relationship between the duration and severity of PD on the one hand, and the cross-sectional area of the vagus nerve on the other (individual mean, calculated from measurements on both sides) in 54 patients. A) Association between the duration of motor Parkinson’s symptoms and the cross-sectional area of the vagus nerve (age-adjusted partial correlation, *p < 0.05; considered non-significant after Bonferroni correction). B) Association between the severity of motor Parkinson’s symptoms as measured by the UPDRS-MDS and the cross-sectional area of the vagus nerve (*p < 0.005; considered significant after Bonferroni correction). C) Association between the severity of non-motor Parkinson’s symptoms as measured by the NMSS and the cross-sectional area of the vagus nerve (p = 0.062)

Ultrasonic mid-cervical VN CSA (Fig. 2C) was bilaterally assessable in 52 patients and 60 controls, and unilaterally (left side only) in the remaining 2 PD patients. The digitized uncommented ultrasound images of 53 VN’s (11 patients and 16 controls) were independently assessed by a rater blinded to diagnoses (JR); inter-rater correlation of CSA measures was high (Pearson correlation, r = 0.82, p < 0.001; 95%CI: 0.70–0.90). VN CSA was smaller in PD patients than in controls (Table 1). In the PD patients, VN CSA (bilateral mean) correlated negatively with motor symptom severity assessed with the MDS-UPDRS-III (partial correlation controlled for age, r = −0.39, p = 0.004; Fig. 4) and, by trend, with motor symptom duration (r = −0.31, p = 0.028). Considering all PD patients with motor symptom duration < 10 years, VN CSA did not differ between patients and controls (1.69 ± 0.58 vs. 1.79 ± 0.49 mm; p = 0.39). In contrary, when considering only body-first PD patients (motor symptom duration < 10 years), VN CSA was however smaller than in controls (1.43 ± 0.41 vs. 1.79 ± 0.49 mm^2^; p = 0.007). Considering only brain-first PD patients (motor symptom duration < 10 years), VN CSA (1.89 ± 0.59 mm^2^) was similar as in controls (p = 0.50). Moreover, VN CSA was smaller in body-first than in brain-first PD patients (Table 2). When only de novo PD patients were analyzed, VN CSA at least tended to be smaller in body-first than in brain-first PD patients (1.64 ± 0.35 vs. 1.95 ± 0.52 mm^2^; p = 0.15; Fig. 3C).

### Correlation between VN CSA and vagal function measures

In patients and controls combined, smaller VN CSA (individual bilateral mean) correlated with larger NMSS sum score (partial correlation controlled for age, r = −0.30, p = 0.001, n = 111), but not with NMSS gastric score (p = 0.17). VN CSA tended to correlate with RMSSD during respiratory sinus arrhythmia maneuver (r = 0.26, p = 0.022, n = 75). When focusing on patients only, smaller VN CSA (individual bilateral mean) also tended to correlate with larger NMSS sum score (r = −0.27, p = 0.062, n = 48; Fig. 4C), but again not with NMSS gastric score (p = 0.20). VN CSA in patients correlated, by trend, with RMSSD during respiratory sinus arrhythmia maneuver (r = 0.27, p = 0.095). Considering body-first PD patients only, smaller VN CSA tended to correlate with larger NMSS sum score (r = −0.49, p = 0.074, n = 15), but not with NMSS gastric score (p = 0.19).

### Correlation between VN CSA and pupillary function measures

Right and left VN CSA tended to correlate with ipsilateral pupil constriction velocity in the control group (partial correlation controlled for age, r = 0.34, p = 0.071) but neither in de novo PD patients (p = 0.34) nor in body-first PD patients (p = 0.28). Individual bilateral mean VN CSA values correlated, by trend, with pupil constriction velocity in the control group (partial correlation controlled for age, r = 0.33, p = 0.097), but not in de novo PD patients (p = 0.56) and in body-first PD patients (p = 0.68). VN CSA was unrelated to other pupillometry findings. In the remaining study participants, who did not undergo pupillary sonography (34 patients, 30 controls), bilateral mean VN CSA did not correlate with the self-perceived degree of over-sensitivity to bright light (item 19 of the SCOPA-OUT) both in controls as well as PD patients (p > 0.3, each).

### Classification of de-novo patients by measurement findings

**Fig. 5:**
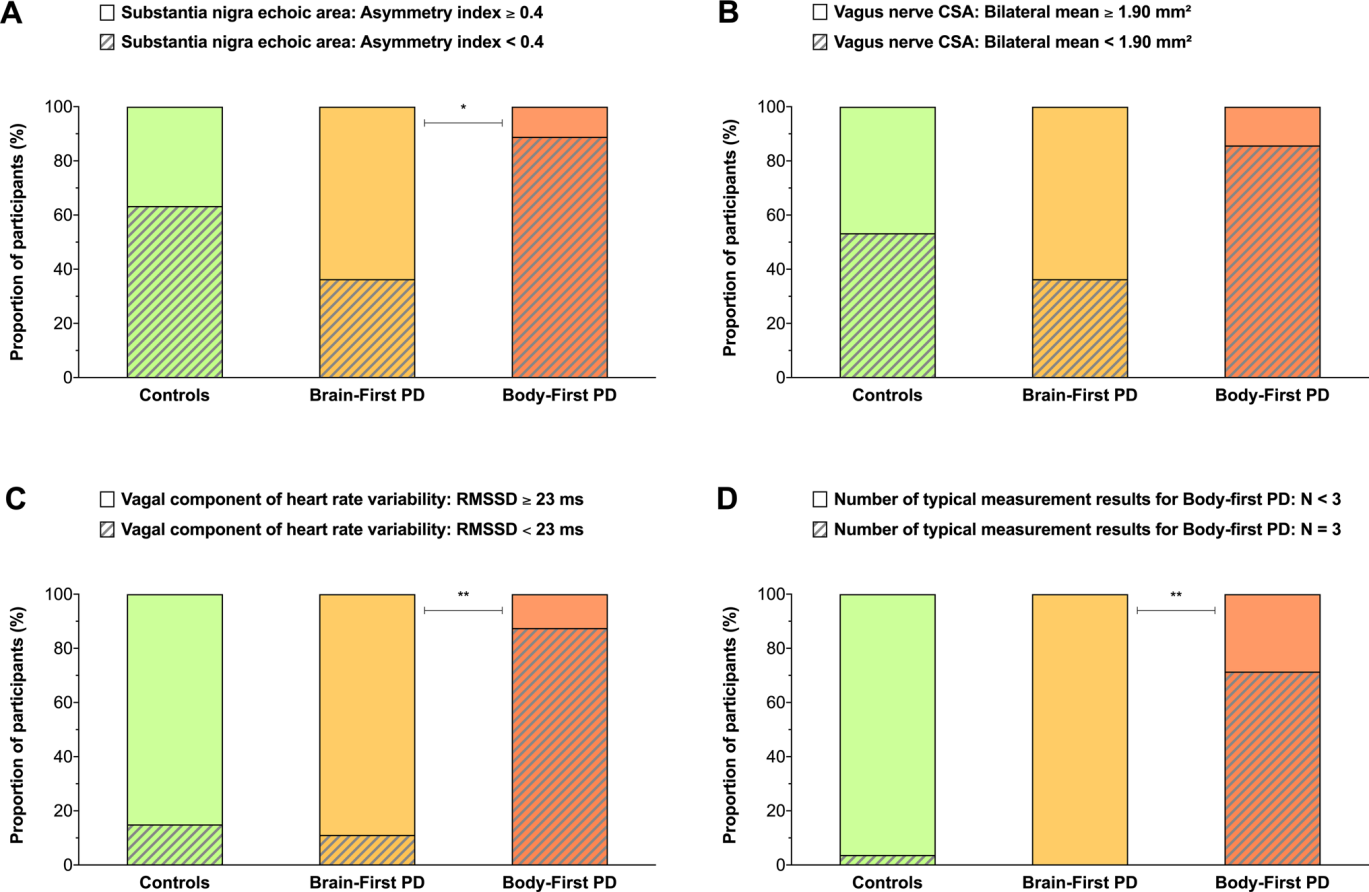
Diagrams showing the prevalence of abnormal findings in supplementary diagnostic tests that suggest brain-first PD, in de novo patients clinically diagnosed with brain-first PD (n = 11) or body-first PD (n = 9), as well as in age-matched control subjects. A) Prevalence of an individually symmetrical echogenicity of the substantia nigra, defined by an asymmetry index < 0.4 for the individual echogenic areas on the right and left (* Fisher’s exact test, p < 0.05). B) Prevalence of an individually reduced cross-sectional area (CSA) of the vagus nerve < 1.9 mm^2^ (individual mean, calculated from measurements on both sides). C) Prevalence of an abnormal vagal component of heart rate variability, defined as the root mean square of successive R-R interval differences (RMSSD) < 23 ms (** p < 0.005). D) Frequency of the simultaneous presence of all three findings indicative of brain-first PD (see panels A, B, and C; ** p < 0.005)

A VN CSA (bilateral mean) of < 1.9 mm^2^ best discriminated body-first from brain-first PD patients (ROC curve analysis, AUC 0.76, 95%CI 0.60–0.92, *J* = 0.51, p = 0.002). RMSSD during respiratory sinus arrhythmia maneuver of < 23 ms best discriminated body-first from brain-first PD patients (AUC 0.80, 95%CI 0.63–0.97, *J* = 0.59, p = 0.001). In de novo PD patients, those classified clinically as body-first PD were indicated (i) by a symmetric SN echogenic area (individual AI < 0.4) with a sensitivity of 88.9% and a specificity of 63.6% (PPV 66.7%, accuracy 75.0%; Fisher’s exact test, p = 0.028); (ii) by a VN CSA < 1.9 mm^2^ (mean of individual bilateral measures) with a sensitivity of 85.7% and a specificity of 63.6% (PPV 60.0%, accuracy 72.2%; p = 0.066); (iii) by RMSSD during respiratory sinus arrhythmia maneuver of < 23 ms with a sensitivity of 87.5% and a specificity of 88.9% (PPV 87.5%, accuracy 88.2%; p = 0.003); and (iv) by the presence of all 3 criteria (i, ii, iii, if all were assessable) with a sensitivity of 71.4% and a specificity of 100% (PPV 100%, accuracy 88.9%; p = 0.002; Fig. 5).

## Discussion

To investigate the’brain-first’ and ‘body-first’ concepts in PD in greater detail, we conducted a simultaneous multimodal ultrasonic examination of the vagus nerve, pupillary function, and the substantia nigra. We thus showed that VN atrophy in PD correlates with (motor) disease duration and severity. In the first decade of motor disease, significant VN atrophy seems to occur in body-first, but not in brain-first PD patients. VN atrophy in PD was unrelated to alteration of parasympathetic pupillary innervation, which could be detected in both de novo brain-first and body-first PD patients. In contrast, mild dysfunction of sympathetic pupillary innervation, as indicated by a smaller scotopic pupil size at baseline, was present in de novo body-first, but not brain-first PD patients. Finally, ultrasonic alteration of substantia nigra was symmetric in body-first PD, but asymmetric in brain-first PD.

The hypothetical concept of two distinct subtypes of PD with (i) a brain-first pattern and top-down progression of misfolded of alpha-synuclein deposition, and (ii) a body-first pattern and down-top progression of neuropathology, is still a matter of debate [42–44]. Moreover, the clinical-, imaging- or biomarker-based definitions of these two PD subtypes are not yet finalized [45]. Since there is the concept of α-synuclein pathology being present early in the enteric nervous system in body-first PD [1–3], we here applied a modified criterion for body-first PD, requiring the presence of pRBD and/or significant gastrointestinal symptoms, each with onset prior to onset of motor symptoms. Previous studies made the clinical definition of body-first PD dependent on the presence of polysomnography-proven RBD, or pRBD diagnosed using standardized questionnaires [29, 33, 46].

The atrophy of the VN in PD, first described in 2018 [10–12], has been confirmed in a number of studies, but has not been observed in all studies since then [14, 47]. Our current observations of a clear correlation between VN atrophy and PD severity, as well as its occurrence in body-first PD (presence of idiopathic RBD before motor symptom onset) rather than brain-first PD, confirm latest pilot findings [48, 49]. These could also explain why no VN atrophy was found in some earlier studies. Our data also agree with the previously reported inverse correlation between VN CSA and electrocardiographic parameters representing parasympathetic modulation by vagal activity [10, 13, 50]. These findings are consistent with more pronounced parasympathetic (cholinergic) denervation in the body-first PD subtype, which has been demonstrated in radiotracer imaging and postmortem histopathological examinations [7, 33].

We present for the first time results obtained using ultrasound-based dynamic pupillometry in Parkinson’s patients. Compared to commonly used infrared and video tracking methods, the ultrasound approach is more widely available and can be particularly useful in clinical settings where infrared/video pupillometers are not available but point-of-care ultrasound devices are [18]. Studies have demonstrated the high validity of ultrasound measurements of pupil function compared to infrared measurements [34, 51]. Dopaminergic medication is well-known to increase pupil diameter at rest and pupil re-dilation velocity in PD patients [52–55], most likely by excitation of alpha-adrenergic receptors at the dilator pupillae muscle [56]. We therefore performed pupillometry only in drug-naïve PD patients. It has previously been shown that scotopic pupil size in PD patients is smaller than in control subjects, although there is overlap between these groups [57, 58]. Our finding of a smaller scotopic pupil size in patients with de novo body-first PD, but not in patients with de novo brain-first PD, provides evidence of an early change in sympathetic ganglion activity in the body-first subtype. This may be due to the involvement of both the sympathetic ganglion stellate cells and the caudal brainstem nuclei. Of the latter, the paragigantocellular nucleus in particular stimulates both sympathetic preganglionic neurons and co-activates the noradrenergic system associated with the Edinger-Westphal nucleus (e.g. the locus coeruleus) [59, 60]. This fits well with previously reported observations of an early, caudocranial progression of changes in the sympathetic nervous system in body-first PD which is characterized, for example, by the onset of REM sleep behavior disorder in the premotor stages of the disease [7, 61–63]. Accordingly, patients with pure autonomic failure who converted to PD or Lewy body dementia (DLB) after a median follow-up period of 13 years typically had normal pupils (with no signs of sympathetic deficits) at the start of the study, during the early premotor stage of the disease [64].

Our finding that baseline pupil diameter correlates with pupil re-dilation velocity is consistent with the idea that baseline sympathetic ganglion activity influences both baseline pupil size and the velocity of pupil re-dilation after light-induced constriction. However, pupil re-dilation velocity does not differ between de novo patients and control subjects, nor between body-first and brain-first PD patients. This suggests that (i) the main factor for pupillary re-dilation, i.e., the rapid decline in pretectal input to the Edinger-Westphal nucleus after the light is turned off [65], remains unaffected in de novo brain-first and body-first PD patients, and (ii) that the mild sympathetic dysfunction in body-first PD patients has no relevant influence on pupil re-dilation at this stage of PD.

On the other hand, a reduction in pupil constriction speed in PD, which was observed in this study, has already been demonstrated previously [66, 67]. Furthermore, it has been shown that the speed of pupil constriction is more severely impaired in later stages of PD [36], which could explain the lack of detection in a study of early-stage PD patients [68]. When using pilocarpine (parasympathomimetic) eye drops, PD patients were found to have a parasympathetic denervation super-sensitivity of the pupils even at an early stage of the disease, which is thought to be due to pathological involvement of the Edinger-Westphal nucleus [69]. Our findings support the assumption that a change in parasympathetic activity, presumably caused by pathological involvement of the Edinger-Westphal nucleus, occurs in both de novo brain-first and body-first PD.

**Fig. 6:**
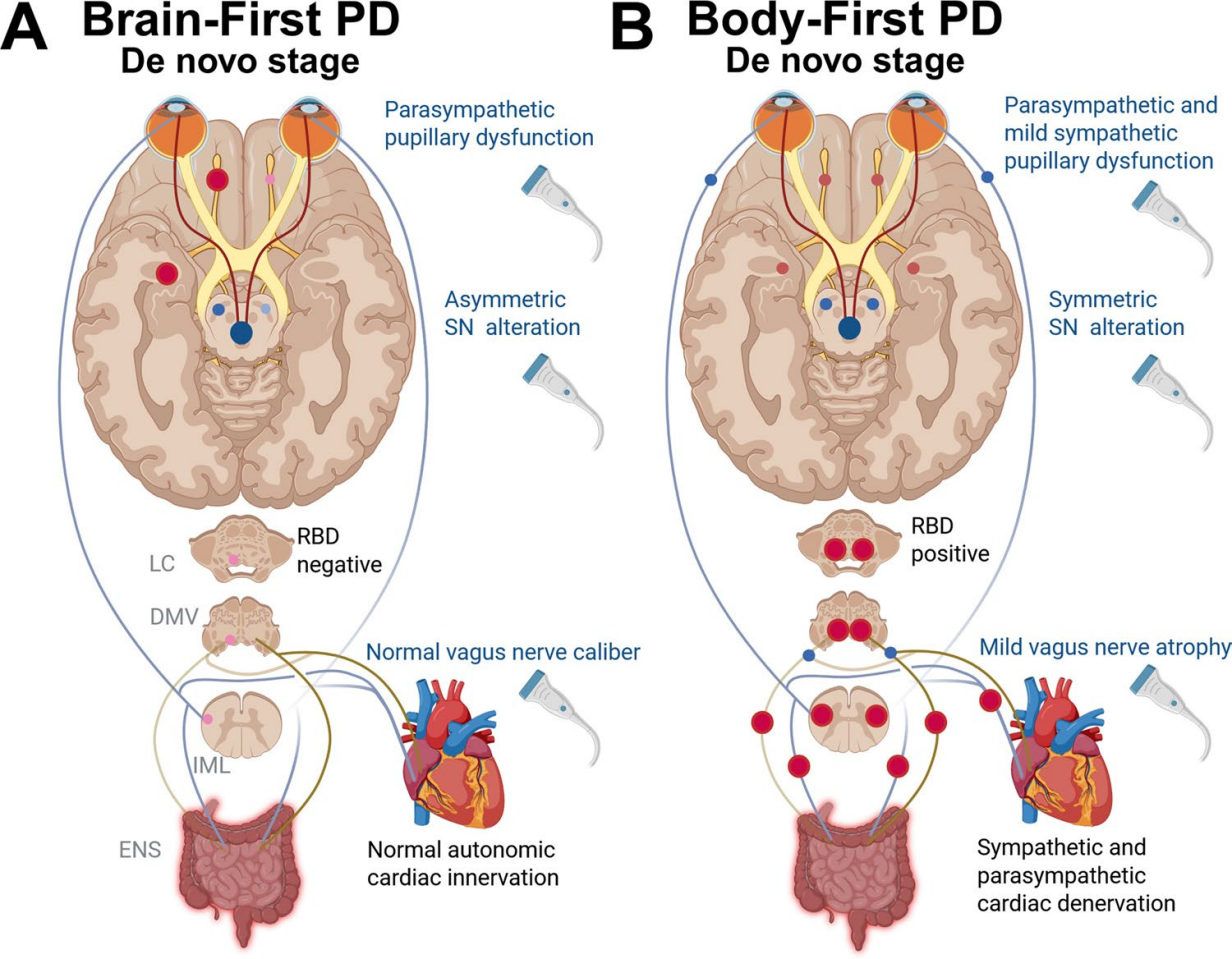
Hypothetical body-first and brain-first subtypes of Parkinson’s disease (modified from Borghammer P, et al. [3]). The figure illustrates the severity of changes in brain structures as well as in the structures of the sympathetic and parasympathetic nervous systems during the de novo stage of PD. Small light dots represent mild changes, medium-sized dots represent moderate changes, and large dark dots represent severe changes. The red dots represent changes previously described, [3] while the blue dots represent the changes shown in this study. **A** Brain-first PD. The initial α-synuclein pathology originates in the olfactory bulb or the amygdala and spreads to closely connected structures. Due to the predominantly ipsilateral connections within a hemisphere, the pathology initially spreads to ipsilateral structures. In the de novo stage, ipsilateral degeneration of the substantia nigra is reflected by an asymmetric echogenic pattern on ultrasound and gives rise to asymmetric motor symptoms. However, the parasympathetic Edinger-Westphal nucleus, located near the midline, is already symmetrically altered. **B** Body-first PD. The initial α-synuclein pathology originates in the ENS and spreads bilaterally to the DMV and IML due to overlapping autonomic innervation. In the de novo stage, there is mild bilateral atrophy of the vagus nerve, although the diameters of the vagus nerve remain within the normal range. The symmetrical α-synuclein pathology spreads rostrally and leads to a more symmetrical change in the substantia nigra, which is reflected in a symmetrical echogenic pattern on ultrasound and more symmetrical motor symptoms. In the de novo stage, there is a mild symmetrical change in sympathetic pupillary innervation as well as a moderate change in parasympathetic pupillary innervation from the Edinger-Westphal nucleus. *DMV* dorsal motor nucleus of vagus, *ENS* enteric nervous system, *IML* intermediolateral cell column, *LC* locus coeruleus, *RBD* REM sleep behavior disorder, *SN* substantia nigra. Made with Biorender.com

A key question that motivated this study was to examine whether the degeneration of the vagal and non-vagal (oculomotor) parasympathetic nuclei in the brainstem, both of which have been demonstrated affected neuropathologically in PD [70–72], occurs simultaneously or not. Our results show that vagal and non-vagal parasympathetic neurodegeneration occur independently of one another. In the stage of de novo PD, oculomotor parasympathetic dysfunction is present in both body-first and brain-first PD patients, but vagal atrophy is present in body-first PD only. This is consistent with the idea that, in the de novo stage, both the presumed top-down neurodegeneration in patients with brain-first PD and the presumed bottom-up neurodegeneration in patients with body-first PD have reached the mesencephalic brainstem, at a stage when the neurons of the substantia nigra are also significantly degenerated resulting in the emergence of motor symptoms (Fig. 6).

The characteristic finding of hyperechoic substantia nigra in PD has been described first in 1995 [73]. Meanwhile the assessment of substantia nigra and basal ganglia on TCS has been established as a diagnostic marker for the early and differential diagnosis of PD [16, 17]. The histochemical correlate of increased echogenicity in the anatomical region of the substantia nigra is likely iron bound to ferritin [37, 74]. The degree of individual right-left asymmetry in echogenicity in the substantia nigra region correlated with the asymmetry of iron accumulation on susceptibility-weighted MRI [37]. The results of numerous studies suggest that the hyperechoic alteration in the substantia nigra is a relatively stable finding in PD, which is present several years before the onset of obvious motor symptoms and remains unchanged as the disease progresses [16]. However, the results of some studies imply that the echogenicity of the substantia nigra in people with PD may change slightly over time [75, 76]. In most patients with PD, the side of the substantia nigra with the larger echogenicity was contralateral to the side of the dominant motor impairment [77, 78], and higher individual right-left asymmetry in substantia nigra echogenicity was related to more pronounced motor asymmetry [78]. According to our present data, the increase of substantia nigra echogenicity is more symmetric in body-first PD, and more asymmetric in brain-first PD. This is consistent with the presumed more symmetrical degeneration of the substantia nigra in in body-first PD, and a rather asymmetrical degeneration in brain-first PD [79]. It has been hypothesized that most patients with dementia with Lewy bodies (DLB) belong to the body-first subtype, whereas patients with PD more often belong to the brain-first subtype, and it was demonstrated that patients with DLB, on average, exhibit significantly more symmetrical degeneration of the striatum than patients with PD [80]. Consistent with this, it has been shown earlier that patients with DLB typically exhibit a symmetrical increase in the echogenicity of the substantia nigra, whereas patients with PD (with or without late-onset dementia) more frequently exhibit asymmetrical echogenicity [81–83]. These results support the hypothesis that the body-first subtype is characterized by a symmetrical degeneration of substantia nigra, whereas the brain-first subtype is related to a more lateralized initial propagation of pathology. Since the echogenicity of the substantia nigra is more of a risk marker (e.g., caused by iron overload) than a sign of degeneration, it remains to be determined whether the sonographic change could represent an early reactive phenomenon triggered by the initial signals of the (asymmetric) top-down or (symmetrical) bottom-up disease process. This concept is supported by the results of postmortem examinations of brains from non-PD patients, which have shown a correlation between increased echogenicity and microglial activation in the substantia nigra [84].

Several weaknesses of our study deserve to be addressed. First, there is currently no consensus regarding the clinical, imaging, biomarker-based, or neurophysiological diagnostic criteria for the presumed brain-first and body-first subtypes of PD. We have applied criteria here that were adapted from previously reported criteria and are based on questionnaires used to assess the likelihood of probable RBD and gastrointestinal dysfunction in the premotor phase of PD. Furthermore, these criteria were applied here to patients with a motor symptom duration of up to 9 years. We therefore cannot rule out the possibility that some of our patients were misclassified, especially since we did not perform either polysomnography or objective assessments of gastrointestinal motility. Again, it should be emphasized that the concept of brain-first and body-first subtypes of PD remains controversial [42–44], and that the existence of a third subtype characterized by multifocal onset of Lewy body pathology has also been considered [85]. Second, the number of de novo patients who underwent pupillary function testing is rather small, due to strict application of the predefined exclusion criteria. Although ultrasonic pupillometry is feasible, it is more time-consuming, particularly with regard to offline data analysis. Future studies involving larger patient groups should preferably use standard devices such as video pupillometers, which facilitate easier pupil detection and data analysis [36]. Third, there was no complete blinding regarding group assignment (patients versus control group) for staff members and family members who were part of the control group. Since the most relevant ultrasound measurements (vagal caliber, pupillary function) were (re)analyzed offline by independent researchers, we are confident that this did not cause any significant bias. Fourth, due to the cross-sectional design of this study, we did not collect longitudinal data on pupillary function, the caliber of VN, or the echogenicity of the substantia nigra in individual patients. Such data would be of interest for further characterizing the dynamics of disease progression in the suspected subtypes of PD. Ongoing follow-up studies at our center are addressing this question, and independent validation of present findings by other centers is desirable.

To summarize, the degenerative processes affecting the VN and the parasympathetic pupillary innervation occur separately in PD. In PD patients classified as the body-first subtype based on clinical criteria, detectable VN atrophy occurs early in the course of the disease, whereas in PD patients classified as the brain-first subtype, VN atrophy may not occur until late stages of the disease. However, changes in parasympathetic pupillary innervation can be detected in both patients with de novo brain-first PD and patients with body-first PD, i.e., when the presumed top-down or bottom-up neurodegeneration has reached the mesencephalic brainstem—at a stage in which the neurons of the substantia nigra have already degenerated significantly, leading to the onset of motor symptoms. The sonographic changes in the substantia nigra may indicate early tissue alteration that, in brain-first PD, is triggered by a more asymmetrical disease process progressing from top to bottom, whereas in body-first PD, it is attributable to a more symmetrical disease process progressing from bottom to top. If these findings are confirmed by long-term and postmortem studies, the combined assessment of the echopatterning of the substantia nigra, the caliber of the VN, and heart rate variability could become a useful tool for distinguishing between the brain-first and body-first subtypes of PD. Short of neuropathological studies, a combination of refined clinical and device-based biomarkers could potentially serve as surrogate indicators for these Parkinson’s subtypes.

## Supplementary Information

Below is the link to the electronic supplementary material.

Supplementary file1 (PDF 337 KB)

## Data Availability

The datasets used and/or analyzed during the current study are available from the corresponding author on reasonable request.
