# Supplementary material for: Brain-first versus body-first Parkinson’s disease: Differential findings on pupillary, brainstem and vagus sonography
Source: J Neurol. 2026 Jul 2;273(8):444. doi: 10.1007/s00415-026-13973-0 (PMC13328145; doi:10.1007/s00415-026-13973-0)
Supplement: Supplementary file 1 — Supplementary file1 (PDF 337 KB) [file 415_2026_13973_MOESM1_ESM.pdf]

Supplementary Material

Brain-first versus body-first Parkinson's disease: Differential findings on pupillary, brainstem and vagus sonography

Uwe Walter, MD, FEAN [1,2]

Michael Batchakaschvili [1]

Hanna Rebekka Kleinlein [1]

Jakub Radziwon [1,3]

Wiebke Hermann, MD [1,2]

Hartmut Walter, MSc [1†]

Alexander Storch, MD [1,2]

Matthias Löhle, MD [1,2]

[1] Department of Neurology, Rostock University Medical Center, Rostock, Germany

[2] Deutsches Zentrum für Neurodegenerative Erkrankungen (DZNE) Rostock/Greifswald, Rostock, Germany

[3] Department of Neurology, Neurodegenerative Disorders and Neuroimmunology, Faculty of Health Sciences, Medical University of Gdańsk, Poland

[†] Deceased.

Corresponding author:
Prof. Dr. Uwe Walter
Department of Neurology
Rostock University Medical Center
Gehlsheimer Str. 20
18147 Rostock, Germany
tel : +49-381-494-9696
fax : +49-381-494-4794
email : uwe.walter@med.uni-rostock.de

Supplementary Table S1. Findings in the different motor subtypes of 54 PD patients

Feature	TD PD	AD PD	RD PD	PIGD PD	P_1	P_2	P_3
Demographics							
Age (years), mean ± SD	68.8 ± 9.2	65.5 ± 8.4	74.0	71.3 ± 10.1	0.28 [a]	0.43 [a]	0.08 [a]
Gender, female/male (N)	4/14	7/10	1/0	9/9	0.29 [b]	0.16 [b]	0.74 [b]
PD duration (motor onset, years)	5.1 ± 4.8	3.8 ± 4.9	19.8	11.7 ± 7.3	0.42 [a]	**0.003** [a]	**<0.001** [a]
Clinical scores							
MDS-UPDRS part III, median [IQR]	37 [26, 43]	24 [20, 33]	87	46 [28, 54]	**0.008** [c]	0.26 [c]	**0.003** [c]
NMSQ sum score, median [IQR]	9 [7, 10]	4 [4, 7]	15	13 [10, 15]	**0.017** [c]	**0.002** [c]	**0.001** [c]
NMSS sum score, median [IQR]	40 [24, 95]	28 [15, 50]	152	58 [41, 89]	0.15 [c]	0.31 [c]	**0.031** [c]
NMSS domain 1 (orthostatic hypotension)	1 [0, 3]	0 [0, 2]	12	0 [0, 3]	0.28 [c]	0.41 [c]	0.95 [c]
NMSS domain 6 (gastrointestinal tract)	0 [0, 14]	0 [0, 5]	16	6 [0, 12]	0.44 [c]	0.37 [c]	**0.036** [c]
NMSS domain 7 (urinary function)	4 [0, 22]	4 [0, 13]	23	10 [0, 25]	0.65 [c]	0.90 [c]	0.45 [c]
NMSS domain 8 (sexual function)	0 [0, 4]	0 [0, 0]	12	0 [0, 0]	0.22 [c]	0.35 [c]	0.64 [c]
Olfactory testing							
SS-16 score, median [IQR] [d]	7 [4, 8]	9 [4, 12]	3	4 [2, 8]	0.31 [c]	0.09 [c]	**0.039** [c]
Heart rate variability (vagal component)							
RMSSD (ms), median [IQR] [e]	18 [0, 26]	28 [10, 31]	18	5 [0, 19]	0.23 [c]	0.19 [c]	**0.020** [c]
Sonography findings							
SN echoic area (mm^2) [f]	0.30 ± 0.08	0.32 ± 0.09	0.43	0.31 ± 0.12	0.68 [a]	0.75 [a]	0.97 [a]
SN, normal/hyperechoic (N) [f]	2/16	4/13	0/1	3/14	0.40 [b]	0.66 [b]	1.0 [b]

SN echoic area, asymmetry index [g]	0.37 ± 0.24	0.41 ± 0.24	0.42	0.43 ± 0.31	0.69 [a]	0.57 [a]	0.83 [a]
SN echoic area, symmetric/asymmetric (N) [g]	12/6	9/8	0/1	10/7	0.50 [b]	0.73 [b]	1.0 [b]
Midbrain raphe echo score, median [IQR] [g]	3 [2, 3]	3 [3, 3]	2	3 [3, 3]	0.24 [c]	0.24 [c]	1.0 [c]
Pupil diameter at baseline (mm) [h]	3.49 ± 0.69	3.49 ± 1.05	-	3.00	1.0 [a]	0.51 [a]	0.67 [a]
Pupil constriction velocity (mm/s) [h]	2.18 ± 0.44	2.22 ± 0.73	-	2.19	0.89 [a]	0.99 [a]	0.97 [a]
Pupil re-dilation velocity (mm/s) [h]	0.87 ± 0.24	1.05 ± 0.46	-	1.20	0.30 [a]	0.23 [a]	0.77 [a]
Vagus nerve CSA (mm^2) [i]	1.59 ± 0.54	1.81 ± 0.52	1.15	1.28 ± 0.48	0.26 [a]	0.08 [a]	**0.004** [a]

AR PD: appendicular dominant PD; CSA: cross-sectional area; MDS-UPDRS-III: motor part of the MDS-sponsored Unified PD Rating Scale; NMSQ: Non-Motor Symptoms Questionnaire for PD; NMSS: Non-Motor Symptoms Score for PD; PIGD PD: postural instability/gait difficulty PD; RD PD: rigidity dominant PD; RMSSD: root mean square of successive differences of R-R intervals on electrocardiogram with 0.2-Hz metronom-guided breathing; SN: substantia nigra; SS-16: 16-item Sniffin' Sticks test; TD PD: tremor dominant PD

P_1: comparison of groups TD PD and AD PD (significant *P* values <0.05 in bold); P_2: comparison of groups TD PD and PIGD PD (significant *P* values <0.05 in bold); P_3: comparison of groups AD PD and PIGD PD (significant *P* values <0.05 in bold)

[a] *t*-test, two-sided
[b] Fisher's exact test
[c] Mann-Whitney *U* test
[d] assessed in 19 brain-first and 15 body-first patients
[e] assessed in 14 brain-first and 15 body-first patients
[f] larger of bilateral measures, SN bilaterally assessable in 19 brain-first and 16 body-first PD patients
[g] for details, see text
[h] assessed in 9 TD PD, 9 AD PD patients, and one PIGD PD patient
[i] individual mean of bilateral measures

Supplementary Table S2. Findings in 20 de-novo PD and 30 age-matched controls

Feature	PD patients	Controls	*P*
Demographics			
Age (years), mean ± SD	64.5 ± 9.2	64.8 ± 6.4	0.90 [a]
Gender, female/male (N)	9/11	14/16	1.0 [b]
PD duration (motor onset, years)	1.5 ± 0.6		
Clinical scores			
MDS-UPDRS part III, median [IQR]	28 [21, 38]	0 [0, 0]	**<0.001** [c]
NMSQ sum score, median [IQR]	6 [4, 9]	3 [2, 7]	**0.014** [c]
NMSS sum score, median [IQR]	27 [13, 38]	11 [3, 25]	**0.006** [c]
NMSS domain 1 (orthostatic hypotension)	0 [0, 3]	0 [0, 0]	**0.023** [c]
NMSS domain 6 (gastrointestinal tract)	0 [0, 6]	0 [0, 0]	**0.017** [c]
NMSS domain 7 (urinary function)	4 [0, 5]	3 [0, 7]	0.81 [c]
NMSS domain 8 (sexual function)	0 [0, 0]	0 [0, 0]	0.40 [c]
Olfactory testing			
SS-16 score, median [IQR] [d]	7 [7, 11]	11 [10, 13]	**0.002** [c]
Heart rate variability (vagal component)			
RMSSD (ms), median [IQR] [e]	23 [14, 29]	42 [24, 64]	**0.002** [c]
Sonography findings			
SN echoic area (mm^2) [f]	0.28 ± 0.08	0.18 ± 0.07	**<0.001** [a]
SN, normal/hyperechogenic (N) [f]	5/15	24/5	**<0.001** [b]
SN echoic area, asymmetry index [g]	0.42 ± 0.23	0.37 ± 0.27	0.46 [a]
SN echoic area, symmetric/asymmetric (N) [g]	12/8	19/11	1.0 [b]
Midbrain raphe echo score, median [IQR] [g]	3 [2, 3]	3 [3, 3]	0.72 [c]
Pupil diameter at baseline (mm)	3.50 ± 0.85	3.81 ± 0.92	0.10 [a]
Pupil constriction velocity (mm/s)	2.23 ± 0.70	2.62 ± 0.91	**0.020** [a]
Pupil re-dilation velocity (mm/s)	0.97 ± 0.40	0.98 ± 0.36	0.94 [a]
Vagus nerve CSA (mm^2) [h]	1.83 ± 0.48	1.90 ± 0.48	0.65 [a]

CSA: cross-sectional area; MDS-UPDRS-III: motor part of the MDS-sponsored Unified PD Rating Scale; NMSQ: Non-Motor Symptoms Questionnaire for PD; NMSS: Non-Motor Symptoms Score for PD; RMSSD: root mean square of successive differences of R-R intervals on electrocardiogram with 0.2-Hz metronom-guided breathing; SN: substantia nigra; SS-16: 16-item Sniffin' Sticks test

[a] *t*-test, two-sided [b] Fisher's exact test [c] Mann-Whitney *U* test

[d] assessed in 19 brain-first and 15 body-first patients

[f] assessed in 14 brain-first and 15 body-first patients

[f] larger of bilateral measures, SN bilaterally assessable in 19 brain-first and 16 body-first PD patients

[g] for details, see text [h] individual mean of bilateral measures

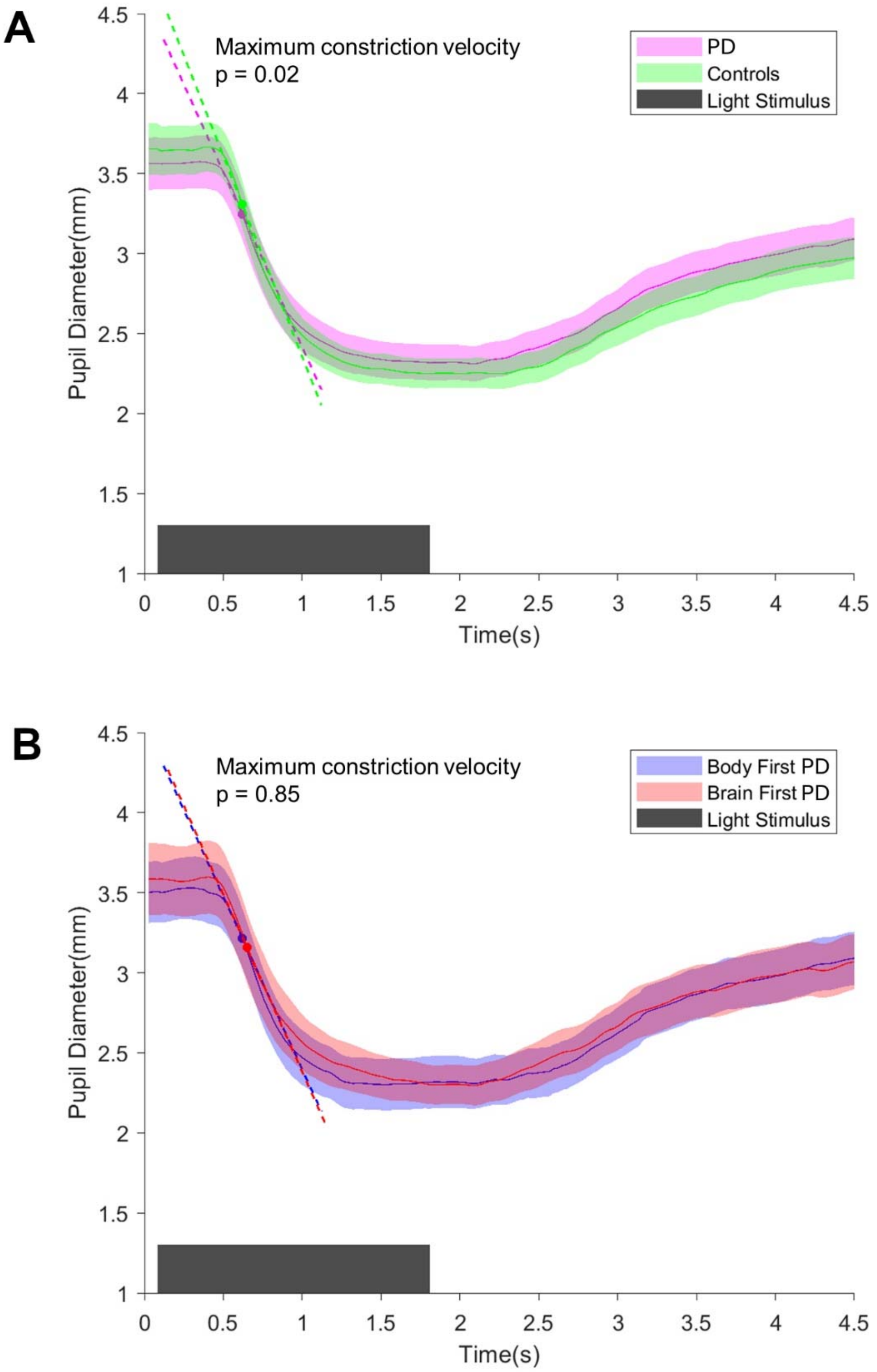

Supplementary Figure S1. Mean pupil light reflex waveforms (mean ± 95% confidence intervals; of bilateral measurements), recorded with ultrasound dynamic pupillometry in 20 drug-naïve de novo PD patents and 30 age-matched control subjects. A) Comparison of pupil light reflex waveforms in PD patients and control subjects. Maximum pupil constriction velocity is significantly reduced in the PD patients. B) Comparison of pupil light reflex waveforms in clinically defined brain-first PD patients (n=11) and body-first PD patients (n=9). Maximum pupil constriction velocity did not differ between these patient groups.

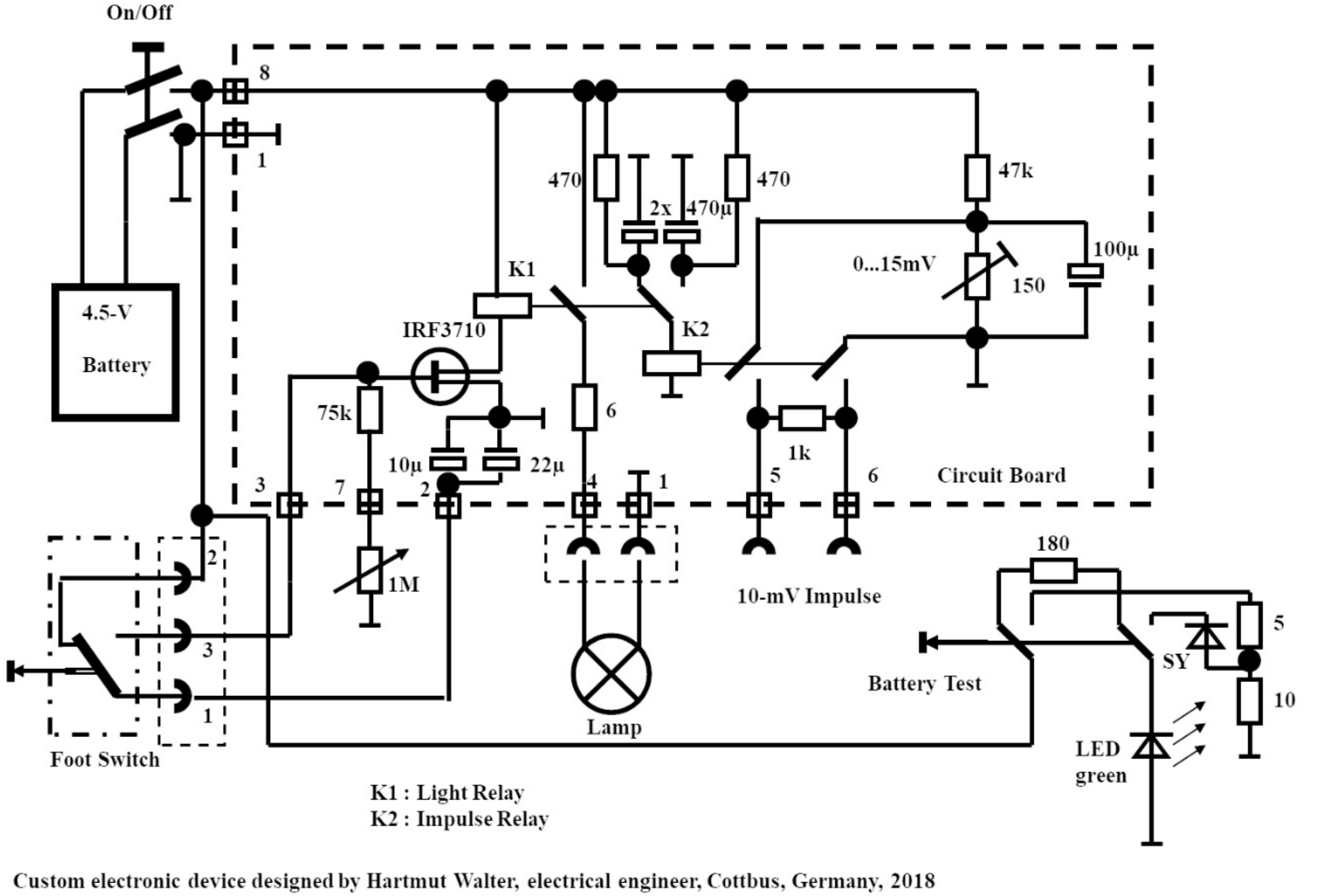

Supplementary Figure S2. Electronic circuit diagram of the custom-made electronic device to control the switching on and off of a commercially available medical penlight used to illuminate the eye for recording of the pupillary light reflex. The impulse relay was designed to deliver signals indicating the timepoints of light-on and light-off switches which were transferred to the ultrasound system via an electrocardiography (ECG) adapter cable, in order to display the light switches on the ultrasound screen (B-mode, M-mode).
